# Patients’ out-of-pocket expenses analysis of presurgical teledermatology

**DOI:** 10.1186/s12962-019-0186-3

**Published:** 2019-08-23

**Authors:** Felipa de Mello-Sampayo

**Affiliations:** Department of Economics, Instituto Universitário de Lisboa (ISCTE-IUL) and BRU-IUL, ISCTE-IUL, cacifo 187, Av. Forças Armadas, 1649-026 Lisbon, Portugal

**Keywords:** Economic analysis, Out-of-pocket expenses, Opportunity cost, Teledermatology, Waiting time

## Abstract

**Background:**

This study undertakes an economic analysis of presurgical teledermatology from a patient perspective, comparing it with a conventional referral system. Store-and-forward teledermatology allows surgical planning, saving both time and number of visits involving travel, thereby reducing patients’ out-of-pocket expenses, i.e. costs that patients incur when traveling to and from health providers for treatment, visits’ fees, and opportunity cost of time spent in visits. to The study quantifies the opportunity costs and direct costs of visits for adults waiting for dermatology surgery.

**Method:**

This study uses a retrospective assessment of 123 patients. Patients’ out-of-pocket expenses of presurgical teledermatology were analyzed in the setting of a public hospital over 2 years. The teledermatology network covering the area served by the Hospital Garcia da Horta, Portugal, linked the primary care centers of 24 health districts with the hospital’s dermatology department. The patients’ opportunity cost of visits and direct costs of visits (transport costs, and visits’ fee) of each presurgical modality (teledermatology and conventional referral), were simulated from initial primary care visit until surgical intervention. Two groups of patients, those with Squamous Cell Carcinoma and those with Basal Cell Carcinoma, were distinguished in order to compare the patients’ out-of-pocket expenses according to the dermatoses.

**Results:**

From a patient perspective, the conventional system was 2.12 times more expensive than presurgical teledermatology. Teledermatology allowed saving €0.74 per patient and per day of delay avoided. This saving was greater in patients with Squamous Cell Carcinoma than in patients with Basal Cell Carcinoma. Although, the probabilistic sensitivity analysis corroborates the results of the base case scenario, only a prospective study can substantiate these results.

**Conclusion:**

In the Portuguese public healthcare system and under specific cost hypotheses, from a patient economic perspective, teledermatology used for presurgical planning and preparation is the dominant strategy in terms of out-of-pocket expenses, outperforming the conventional referral system, especially for patients with severe dermatoses.

## Background

Time spent seeking healthcare represents a burden to patients, lost productivity to employers and society, and a potential inefficiency within healthcare systems. Opportunity costs, which value patient time based on the value of forgone activities are one method of estimating patient time costs of visits [1, 2]. Opportunity costs are increasingly relevant given the increasing emphasis on patient-centered care [3], and the recognition that telemedicine in healthcare delivery options may reduce patients’ burden regarding time and expenses.

One of the specialties in telemedicine, teledermatology (TD) appears as a way to implement dermatological healthcare to underserved areas and populations. Teledermatology may be achieved by videoconference or store-and-forward. In the former, videoconference equipment is used to connect a patient with a remote consultant. In store-and-forward, specialists assess a transmitted still image. Using teledermatology patients do not have to visit the dermatologists physically. By avoiding the need for clinic-based visits, teledermatology also saves on societal costs that are associated with patients’ travel and workplace absenteeism [4, 5]. Teledermatology not only decreases appointment waiting times and the amount of time needed for a consultation, but also reduces transportation costs and loss of productivity [6]. While most literature reports fewer in-person appointments, teledermatoogy can increase the overall appointment burden for some patients. This depends on the type of teledermatology (videoconference or store-and-forward) and on the health system in which it is implemented [7, 8].

Poor health outcomes can result if at least one of the following condition is met: the waiting time for dermatology treatment increases, costs of visits (opportunity costs and direct costs) increase, the patient’s health deteriorates [9–12], the patient tends to withdraw from treatment because (s)he cannot afford the cost of visits [13–16]. Teledermatology reduces the negative effect(s) associated with these risks. Especially for chronic patients, patient-assisted follow-up care at home avoids traveling to a physician and long appointments during work time.

Teledermatology has been shown to be more effective in the management of circumscribed and tumoral lesions than in patients with generalized dermatoses [17]. In patients with skin cancer, store-and-forward teledermatology has been shown to be an effective triage tool that reduces the time to an initial intervention in the specialized dermatology service [18–23]. Presurgical teledermatology using a store-and-forward system may establish a correct diagnosis and even obtain sufficient information to plan a surgical intervention [24, 25]. Consequently, in the field of surgical dermatology, teledermatology offers added value as a complementary tool for the assessment and presurgical management of patients.

In the context of the regional hospital setting and from patients’ perspective, this study seeks to compare out-of-pocket expenses between patients whose routine care was carried out using a store-and-forward teledermatology system and conventional referral system. It identifies and simulates out-of-pocket expenses borne by presurgical dermatology patients. Out-of-pocket expenses refers to the direct payment of money for seeking healthcare. This comprises the direct costs of visits such as transport costs and visit fees, and the opportunity cost of visits, including the time away from paid work, devoted instead to visiting the primary care provider (PCP) or hospital. This study also quantifies the out-of-pocket expenses per day of wait time for presurgical teledermatology when compared to the conventional referral system.

## Methods

The teledermatology network covering the area served by the Hospital Garcia da Horta in Almada, Portugal, links the primary care centers (PCP) of 24 health districts with the hospital’s dermatology department via the corporate intranet of the Portuguese healthcare system. Store-and-forward teledermatology is currently being used as a complementary tool for the triage of patients and the management of patient referral from the primary care center to the hospital in the Portuguese National Health System. Following the first visit to a general practitioner (GP) at PCP, digital pictures of patients who agreed to store-and-forward teledermatology were taken and transmitted to the hospital via intranet. Alternatively some patients were referred to a dermatology visit at the hospital. In total, 153 patients were treated but 30 did not require surgical intervention. This study therefore uses a sample of 123 presurgical cases (falling between February of 2016 and January of 2018). A retrospective assessment was made of the clinical course of 53 patients who were managed with hospital’s dermatologist visits and 70 patients who were managed with store-and-forward teledermatology from initial primary care consultation until the surgical intervention.

### Activity map for Surgical Intervention

**Fig. 1:**
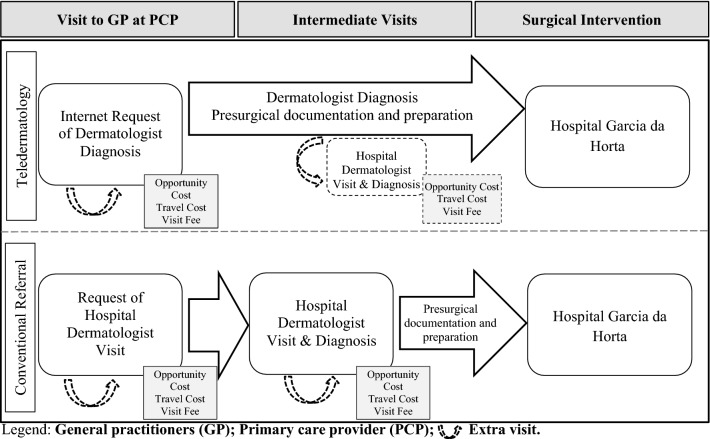
Activity map for surgical intervention of teledermatology and conventional referral systems. GP: General practitioners; PCP: primary care provider; extra visit

The first step was to map all of the activities involved in the process until surgical intervention (see Fig. 1). This study focuses on the patients’ out-of-pocket expenses, and the costs of neither the direct health care expenditures (health care procedures and interventions) nor the indirect health care costs (telecommunications, information technology, and digital photography equipment) were included in the analysis. All patients visited their GP at the PCP. As shown in Fig. 1, patients who were managed with store-and-forward teledermatology needed to visit only PCP before surgical intervention, while those who were managed with conventional care had to visit both PCP and hospital before surgical intervention.

On the basis of this activity map, a specific cost was assigned to each visit involved in the process. Patients’ out-of-pocket expenses of a visit included visit fees, the cost of travel, and the opportunity cost associated with wages lost during the visit. There were 15 patients using teledermatology who were called for an extra visit to the hospital before surgical intervention, and four patients using conventional care who were called for an extra visit. This is illustrated in Fig. 1 by the dashed arrow.

Since one of the stated objectives of the teledermatology system is to reduce time to surgery, two subgroups were also analyzed. The first of these included the patients with lesions suspected of being malignant (Squamous Cell Carcinoma and Melanoma) and the second comprised those who presented the most common lesion among the patients under study (Basal Cell Carcinoma). This comparison allows us to analyze the importance of reduction of time to surgery according to skin lesions.

### Data

Table 1 shows the input data collected from the retrospective assessment of the clinical course of 123 patients. Number of visits corresponds to the total visits the patient made between the initial primary care consultation and the surgical intervention. For both consultation types, wait time was defined as the number of calendar days that elapsed between the initial primary care consultation and the surgical intervention. The distance in kilometers (km), based on patients’ zip code, was used in order to calculate in google maps the travel distance from home to the PCP and to the hospital. Patients’ ages ranged between 22 and 94 years old. This study therefore refers to working and retired adults.

**Table 1:** Input data

	Total patients (123)				Presurgical TD patients (70)				Presurgical CR patients (53)			
	Mean	Stdev	Min	Max	Mean	Stdev	Min	Max	Mean	Stdev	Min	Max
Number of visits	1.59	0.56	1	3	1.21	0.41	1	2	2.08	0.27	2	3
Wait time (days)	103.33	73.14	9	435	86.09	57.62	9	309	126.11	84.95	11	435
Distance to PCP (km)	3.84	5.70	1.00	37.70	3.82	4.31	1.00	22.50	3.86	7.18	1.00	37.70
Distance to hospital (km)	12.79	20.48	1.20	163.00	10.58	9.07	1.20	44.50	15.72	29.33	1.20	163.00
Age	68.64	14.72	22	94	67.37	15.20	25	94	70.32	14.02	22	93
Gender	Men (62); Women (61)				Men (35); Women (35)				Men (27); Women (26)			

TD: Teledermatology; CR: conventional referral; PCP: primary care provider

## Key assumptions

The exact means of transportation that patients used for traveling to visits was not available; as a result it was proxied by taxi since anyone could use a taxi to travel 1 km or 20 km, from city center or from suburbs, allowing a comparison of transport costs among patients. The travel cost was based on the official published fares for Portuguese Taxi transportation [26] by both kilometer and day fare, which was then multiplied by travel distance in kilometers.

The estimation of the opportunity costs for adults was based on mean wage and time spent on visits. Total time per visit comprised travel time and visit time to PCP or to hospital. Employers in Portugal do not pay for time not worked and spent in PCP or hospital visits. Retirement-age patients (≥ 70) were not assigned an opportunity cost since they do not work. Neither the specific wage nor the employment situation were available for the individual patients. As a result, calculating the opportunity cost of visits, i.e. loss of pay during the visits was based on the Portuguese average wage [27]. This study used an average 121 min for total time per visit (with 37 min of travel time and 84 min of clinic time including both waiting and face-to-face time), estimated for ambulatory medical care [28].

All patients in the study are in the public national health system (NHS) and are assumed to pay the regulated basic visit fees (as per Directive no. 64-C/2016, Diário da República no. 63/2016) [29]. The basic fee of visiting general practitioners (GPs) at PCP is €4.5 and the fee for a visit of a specialist at the hospital is € 7.0. For the teledermatology diagnosis, this study used the basic fee of a hospital’s visit without patient (€2.5).

Wait time was defined as the number of calendar days that elapsed between the initial primary care consultation and the surgical intervention. Taking into account the mean wait times for surgery in both presurgical TD and the conventional referral system reported in Table 1, the out-of-pocket expenses per day of wait time saved was calculated as the difference in out-of-pocket expenses relative to the difference in wait time between presurgical teledermatology and convention referral.

## Results

The results of the identification of out-of-pocket expenses are shown in Table 2. Total patients’ mean cost of travel was €23.78. Presurgical TD patients paid €14.31 and presurgical CR patients paid €36.29 in travel costs. The difference in the cost of travel between the opportunity cost of using presurgical TD and using the conventional process was large and significant (P < 0.001). The mean opportunity cost of visits was €10.74 to all patients analyzed. The mean opportunity cost of visits was €15.39 to patients in the conventional referral system and €7.21 in the presurgical. Significant differences were found between the opportunity cost of presurgical modalities (P < 0.001). The mean visit fee was €10.02. There was a difference of €4 between the modalities’ fees, which was found to be significant (P < 0.001).

**Table 2:** Out-of-pocket expenses per patient identification analysis

	Total patients (123)				Presurgical TD patients (70)				Presurgical CR patients (53)			
	Mean	Stdev	95% CI		Mean	Stdev	95% CI		Mean	Stdev	95% CI	
			Low	High			Low	High			Low	High
Opportunity cost of visits (€)	10.74	13.78	8.30	13.17	7.21	8.66	5.19	9.24	15.39	17.54	10.67	20.11
Direct costs of visits												
Visit fees (€)	10.02	3.05	9.48	10.56	8.50	2.89	7.82	9.18	12.03	1.87	11.53	12.53
Transport cost (€)	23.78	26.74	19.05	28.50	14.31	9.81	12.01	16.60	36.29	35.64	26.69	45.88

TD: Teledermatology; CR: conventional referral; PCP: primary care provider

Table 3 shows the detailed out-of-pocket expenses of patients managed by either presurgical TD or the conventional referral system until the surgical intervention. The first column shows the results for all patients and the results for the two subgroups analyzed: patients with Squamous Cell Carcinoma and with Basal Cell Carcinoma are shown in the second and third columns, respectively.

**Table 3:** Out-of-pocket expenses per patient

Presurgical modality	All		Squamous cell carcinoma^a^		Basal CELL carcinoma	
	Presurgical TD patients	Presurgical CR patients	Presurgical TD patients	Presurgical CR patients	Presurgical TD patients	Presurgical CR patients
Opportunity cost of visits (€)	7.21	15.39	3.53	6.36	10.22	14.71
Direct costs of visits						
Visit fees (€)	8.50	12.03	13.46	54.47	17.80	39.18
Transport cost (€)	14.31	36.29	9.33	11.50	9.95	12.38
Out-of-pocket expenses (€)	30.02	63.71	26.33	72.33	37.97	66.27

TD: Teledermatology; CR: conventional referral; PCP: primary care provider

^a^Includes patients with Melanoma

The ratio between the two modalities shows that for all patients, conventional care was 2.12 times more costly to the patients than presurgical TD. In the group of patients who had Squamous Cell Carcinoma, conventional care was 2.75 times more costly, while, in the group of those with Basal Cell Carcinoma, conventional care was 1.75 times more expensive than presurgical TD.

### Sensitivity analysis

**Fig. 2:**
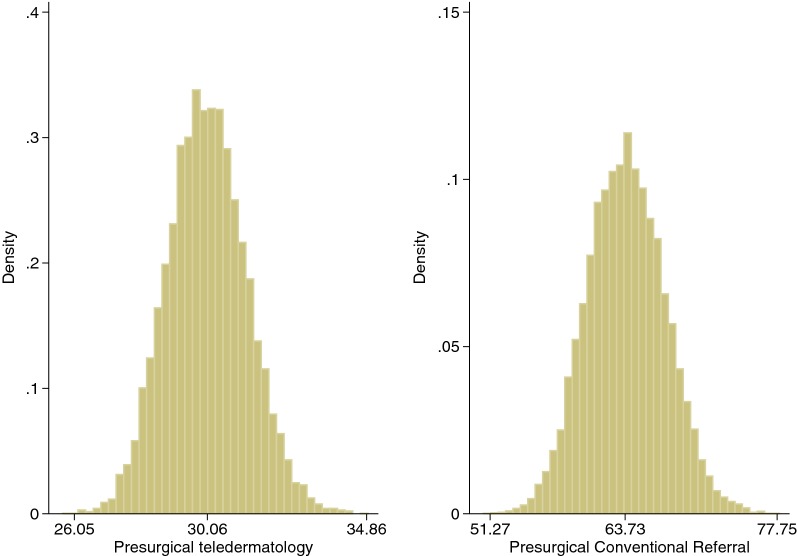
Probability sensitivity analyses of out-of-pocket expenses

To test the robustness of our cost analysis, sensitivity analysis was carried around the assumed transport costs, as taxis are often considerably more expensive than public transport or self-driving. Sensitivity analysis was carried out on the opportunity cost as the wage rates and employment situations were not available; and on visit fees, as patients that are exempted from paying basic fees, such as pregnant women and the unemployed were not considered. The ranges used for the Probability Sensitivity Analysis are reported in Table 4. Parameters were assigned a distribution according to the methodology suggested by Briggs et al. [30]. Those authors suggest using the Gamma distribution for costs where parameters are non-negative. The results of the base case scenario were confirmed (see Fig. 2) after 10,000 simulation draws of the mean of the out-of-pocket expenses.

**Table 4:** Parameters for the probability sensitivity analysis

	Presurgical TD patients (70)					Presurgical CR patients (53)				
	Gamma Dist.^a^	Mean	Stdev	Range		Gamma Dist.^a^	Mean	Stdev	Range	
				Min	Max				Min	Max
Opportunity cost of visits (€)	γ (0.69,0.10)	7.33	8.89	0.00	103.4	γ (0.77,19.98)	15.26	17.01	0.00	184.8
Direct costs of visits										
Visit fees (€)	γ (8.63,1.02)	10.02	3.05	1.51	26.29	γ (41.52,0.29)	12.03	1.87	5.97	20.18
Transport cost (€)	γ (2.13,0.15)	23.78	26.74	0.09	80.69	γ (1.04,35.00)	36.02	35.23	0.00	305.3

^a^The parameters used in the Gamma distribution: $\gamma \left( {\alpha ,\beta } \right)$ where $\alpha = Mean^{2} /Stdev^{2}$ and $\beta = Stdev^{2} /Mean^{2}$ using the mean and variance of the population under analysis

### Out-of-pocket Expenses per day of wait time saved

Table 5 shows that presurgical TD was found to be a better strategy than the conventional consultation process, with a saving of €0.74 per patient and per day of wait time avoided. This saving was much greater for patients with Squamous Cell Carcinoma than for patients who had Basal Cell Carcinoma (€4.35 compared to €0.38).

**Table 5:** Out-of-pocket expenses and time of treatment analysis

Presurgical modality	All patients		Squamous cell carcinoma		Basal cell carcinoma^a^	
	Presurgical TD patients	Presurgical CR patients	Presurgical TD patients	Presurgical CR patients	Presurgical TD patients	Presurgical CR patients
Out-of-pocket expenses (a) (€)	26.34	55.85	26.33	72.33	37.97	66.27
Wait time (days) (b)	86.09	126.11	66.22	76.80	91.74	166.08
Delta out-of-pocket expenses (∆a) (€)		− 29.5		− 46.0		− 28.3
Delta wait time (days) (∆b)		− 40.03		− 10.58		− 74.35
Out-of-pocket expenditure per Wait Time Saved (∆a/∆b) (€)		0.74		4.35		0.38

TD: Teledermatology; CR: conventional referral; PCP: primary care provider

^a^Includes patients with Melanoma

## Discussion

The economic analysis provides information from a series of patients whose routine care was carried out using a store-and-forward teledermatology system and conventional referral system for presurgical assessment in a Portuguese public healthcare setting equipped with intranet.

In the context of the regional hospital setting and the patients’ perspective adopted in the analysis, this study shows store-and-forward teledermatology to be an economically advantageous method for the patients involved in the presurgical assessement and management. Considerable differences were found between the out-of-pocket expenses using presurgical TD and the conventional process. Overall, presurgical TD was 2.12 times less costly than the conventional referral system for patients having surgical intervention (€30.02 per patient compared to €63.71 per patient). Table 3 also shows that the expenses fell substantially among patients with Squamous Cell Carcinoma, making presurgical TD 2.75 times less costly than conventional care (€26.33/patient compared to €72.33/patient). For patients with Basal Cell Carcinoma, presurgical TD is 1.75 times less costly than conventional care.

In the 123 cases in this study, the mean age of the patients was 68.64 years (95% confidence interval [CI], 66.04–71.24 years; range 22–94 years), and there were approximately the same number of men (50.4%) as women (49.6%), see Table 1. The observed difference between the sample means is not considerable to say that the average age and gender between presurgical TD and conventional referral patients differ. Patients who were managed using teledermatology made on average one visit before surgical intervention. Presurgical CR patients made on average two visits before surgery. The mean time to surgical intervention for the patients managed by presurgical TD was 86.09 days. In the group managed using the conventional process, mean time to surgery was 126.11 days.

There were 15 patients using teledermatology who were called for an extra visit to the hospital before surgical intervention, and four patients using conventional care who were called for an extra visit. Systematic reviews show that there is good diagnostic agreement when comparing a teledermatology diagnosis and in-person clinical diagnosis or histopathology with traditional face-to-face consultations [31]. However, several factors may directly impact the reliability of teledermatology, including proper imaging, comprehensive relevant history, and skills of the teledermatologists and referring physicians [32]. The difference of extra visits between the two systems could reflect the fact that a lack of sufficient information to plan the surgical intervention is more frequent in teledermatology than in the conventional method.

The economic results of the use of teledermatology have been analyzed in earlier studies [2, 4–6, 25, 33]. From the point of view of out-of-pocket expenses, there has been no prior analysis of the use of teledermatology in presurgical assessment and management. The economic analysis of presurgical teledermatology in patients with nonmelanoma skin cancer by Ferrándiz et al. [25] found the conventional system to be 1.78 times more expensive than presurgical teledermatology. However, comparisons to their results should not be made because their travel costs took into account the type of transportation used (public, private, or medical transport) and the cost incurred through loss of wages used the minimum wage. Also, their study included direct and indirect healthcare costs.

The expenses relative to wait time difference suggested a €0.74 saving per patient and per day of wait time avoided for patients using presurgical TD. This saving was substantially greater for patients with Squamous Cell Carcinoma and lower for patients with Basal Cell Carcinoma (€4.35 and €0.38 per patient and per day of wait time avoided, respectively). The saving was greater among patients with Squamous Cell Carcinoma because this calculation is based on the reduction in wait time. Patients with Squamous Cell Carcinoma generally required close medical follow-up, giving rise to a reduction in the difference of waiting times between the two modalities, decreasing the mean wait time difference. Furthermore, two patients with Squamous Cell Carcinoma under presurgical TD system required an extra visit to the hospital, which made it more expensive, and thus closer to conventional care.

The study has several limitations, the most important of which concerns the quality of data entered into the model. It was assumed that all patients traveled by taxi, and some patients may have traveled by other means of transportation, namely bus or private transport. This may overestimate the transport cost, as taxis are an expensive means of transportation when compared to public or private transport. Patients who had difficulties in traveling to the hospital (bedridden patients and those in other incapacitating situations) and who required home treatment and medical transport were not taken into account. This may underestimate the travel cost. Companions of patients were not taken into account in the calculation of the expense associated with travel and lost wages. This may underestimate the out-of-pocket expenses. Unemployed and or chronically ill patients were not taken into account when calculating the opportunity cost of visits. This may have overestimated the opportunity cost. The real patient salaries and exact time spent traveling to, from, and during visits were not available, so the opportunity cost was calculated based on average wages and average time spent on visits, which weakens the validity of the results. Finally, the allocation of patients to the subgroups was not random and, therefore one has to be attentive to the potential bias in the analysis between subgroups. Despite these drawbacks, which are typical of most model-based economic evaluations, our study helps to clarify the often contradictory research in the field of economic evaluation of teledermatology.

### Future research

This study would benefit from the greater accuracy of data that a prospective study would afford. In particular, a prospective study would allow knowing how to quantify the value of time, especially for the unemployed and retired patients. It would also allow studying the impact of teledermatology on the quality of life and the quality of care attached to teledermatology as perceived by the patients.

## Conclusion

The results of this study suggest that in the Portuguese public healthcare system and under specific cost hypotheses, store-and-forward teledermatology applied to the preparation and presurgical planning is the dominant strategy in terms of out-of-pocket expenses, outperforming the conventional face-to-face process.

## Supplementary information

**Additional file 1.** Raw data (Table A) and analysed data (Table B) used to estimate out-of-pocket expenses.

## Data Availability

The data used to estimate the out-of-pocket expenses are available in the Additional file 1: [Datarepository.xlsx].
